# Salivary Metabolomics for Diagnosis and Monitoring Diseases: Challenges and Possibilities

**DOI:** 10.3390/metabo11090587

**Published:** 2021-08-31

**Authors:** Eelis Hyvärinen, Minttu Savolainen, Jopi J. W. Mikkonen, Arja M. Kullaa

**Affiliations:** 1 Department of Oral Medicine, Institute of Dentistry, School of Medicine, Kuopio Campus, University of Eastern Finland, FI-70211 Kuopio, Finland; eelishyv@student.uef.fi (E.H.); mints@student.uef.fi (M.S.); 2 SIB Labs Infrastructure Unit, Kuopio Campus, University of Eastern Finland, FI-70211 Kuopio, Finland

**Keywords:** saliva, metabolomics, mass spectrometry, NMR spectrometry, oral disease, systemic disease

## Abstract

Saliva is a useful biological fluid and a valuable source of biological information. Saliva contains many of the same components that can be found in blood or serum, but the components of interest tend to be at a lower concentration in saliva, and their analysis demands more sensitive techniques. Metabolomics is starting to emerge as a viable method for assessing the salivary metabolites which are generated by the biochemical processes in elucidating the pathways underlying different oral and systemic diseases. In oral diseases, salivary metabolomics has concentrated on periodontitis and oral cancer. Salivary metabolites of systemic diseases have been investigated mostly in the early diagnosis of different cancer, but also neurodegenerative diseases. This mini-review article aims to highlight the challenges and possibilities of salivary metabolomics from a clinical viewpoint. Furthermore, applications of the salivary metabolic profile in diagnosis and prognosis, monitoring the treatment success, and planning of personalized treatment of oral and systemic diseases are discussed.

## 1. Introduction

Salivary metabolomics is a rapidly evolving discipline aiming to obtain new biological information about both body health and different oral diseases. Saliva as an important biofluid provides a window into biological pathways in the human body because it contains many of the same biomarkers as blood and serum. A biomarker is defined as “any substance, structure, or process that can be measured in the body or its products and that can influence or predict the incidence of outcome or disease” [1]. The components of interest tend to be at a lower concentration in saliva and their analysis demands more sensitive techniques. Salivary metabolomics, one of the “omics” techniques, opens a “new world” to understand many physiological and pathophysiological processes in this unique organ of the human body, the oral cavity. Salivary metabolomics has many promising possibilities in multiple fields, including medicine, oral medicine, dentistry, sports medicine, toxicology, pharmacology, microbiology, nutrition, and forensic science. However, we have many challenges, because the oral cavity is a very complicated organ, and there are many factors that influence the salivary metabolic profile.

This mini-review article aims to highlight challenges and possibilities of salivary metabolomics from a clinical viewpoint as well as to discuss applications of the salivary metabolic profile in diagnosis and prognosis, monitoring the treatment success, and planning personalized treatment of oral and systemic diseases.

## 2. Saliva in the Oral Defense

Saliva is secreted from three paired major salivary glands (i.e., parotid, submandibular, sublingual) and numerous minor salivary glands, which are located throughout the oral cavity. Saliva formation occurs in two steps: the main components of saliva are produced by the secretory (acinar) cells as primary saliva, which is then modified by the ductal cells. The ductal modification depends on the secretion rate and induces changes in the composition of whole-mouth saliva [2]. The secretion functions of salivary glands are controlled by the autonomic nervous system, by both sympathetic and parasympathetic systems [2,3].

The main function of saliva is to moisten and lubricate surfaces of the oral cavity, pharynx, and esophagus. Whole-mouth saliva (WMS) is a complex biofluid containing many defense mechanisms, which are constituted of secreted products derived from serum, salivary glands, gingival fluid, mucosal transudate, and oral microbes. Saliva with its components forms a protective pellicle both on teeth and oral mucosa [4].

Highly viscoelastic and adhesive mucus, named the mucosal pellicle, together with salivary proteins protects the oral mucosa, which covers roughly 80% of the mouth’s surface area. The mucosal pellicle attaches itself to oral epithelial cells, forming a superficial layer on the oral mucosa [4]. Some protective salivary proteins concentrate on the mucosal surface through specific interaction forming the mucosal pellicle, which could create an immune barrier against oral microbes. The mucosal pellicle forms an immunological reservoir of the oral cavity, and it protects oral surfaces against several microorganisms [4,5,6].

## 3. Salivary Metabolomics in Oral Diseases

**Figure 1:**
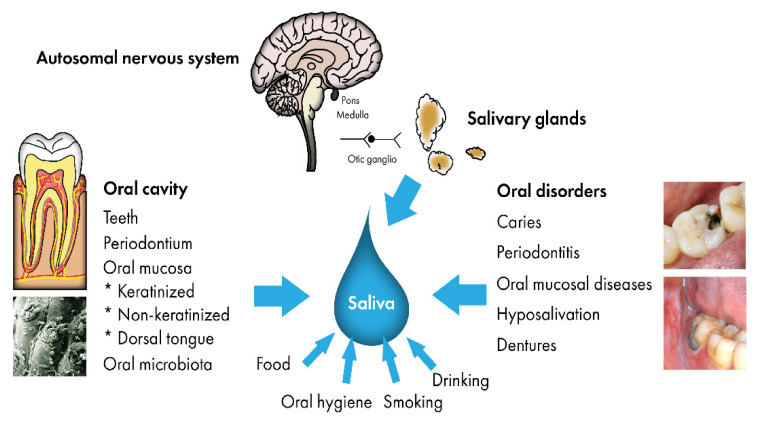
A summary of factors that take part in metabolic pathways in the mouth.

The metabolic pathways of this unique organ are dependent on genetics, proteomics, microbiota, age, gender, environmental alterations, diet, unhealthy habits, systemic diseases, medication, oral diseases, dental materials, dentures, physical training, stress, and hormonal status (endocrine-related metabolites) (Figure 1). Low salivary flow rate can affect the salivary metabolome, thus modifying the salivary composition [2,7]. 

The microbial consortia present in the mouth are associated with human physiological functions including metabolism [8]. The oral cavity contains from 250 to 300 species of micro-organisms forming the oral microbiome, which is considered to maintain homeostasis with many salivary protease inhibitors in a healthy individual [6]. Furthermore, the oral cavity contains many habitats for microbial communities, including teeth, tongue, cheeks, and gums. The dorsal surface of the tongue contains filiform papillae with rough hairs covered by many micro-organisms (Figure 1), and tongue colonization is most important in the healthy mouth. However, quite common and normal tongue disorders—including fissured tongue, geographic tongue, hairy tongue, and filiform atrophy—may modify the salivary metabolic profile. These disorders are not mentioned in any publications of salivary metabolic studies.

For the most part, salivary metabolomic publications have concentrated on periodontitis and oral cancer (Table 1). The most common human illnesses are caries and periodontitis, which are chronic inflammatory diseases and manifest from slow progression. The standard diagnostics are based on visual and morphological changes associated with those diseases, but they have limitations in early prediction. Generalized periodontitis identified in the saliva and salivary metabolic profile could be used to predict this disease [9,10,11,12,13,14,15,16,17,18]. However, the same molecular mediators of oral dysbiosis are linked to cardiovascular diseases [19].

## 4. Salivary Metabolites and Systemic Diseases

Saliva is derived from blood and it reflects the physiological status of the body [29]. Many systemic diseases, medication, and hormones, including insulin, melatonin, estrogens, and androgens, modulate salivary gland function [30]. Salivary metabolites have been studied in the early diagnosis of cancers, but also in neurodegenerative diseases (Table 2). 

## 5. Saliva Collection 

The most critical steps in salivary metabolic experiments are sample collection and preparation preceding the metabolic analysis. Saliva collection is an important issue since different types of saliva have been used in metabolic analysis, whole saliva (WS) is the most used (Table 1 and Table 2). In detail, saliva collection and processing can significantly influence its composition and affect the results. For example, the composition of stimulated saliva differs from that of unstimulated saliva, and masticatory stimulated saliva is verified as an adequate alternative to unstimulated saliva for microbiome-related studies [47]. Therefore, the collection plan depends on the hypothesis of the study.

The collection of saliva is painless, cheap, and easy without risk of infection. It is also suitable for patients who suffer from anxiety during blood collection, and for children and patients with impairments in social communications, including autistic disorder and dementia. Saliva is a complex mixture, therefore optimal sampling methods and storing procedures of saliva samples are important pre-analysis criteria. However, there are many challenges in saliva collection for metabolomic analysis.

Although saliva is easy to collect, many patients suffer from hyposalivation and low masticatory function. Hyposalivation is common in geriatric patients and patients with various chronic diseases and conditions, e.g., Sjögren’s syndrome, rheumatoid arthritis, diabetes mellitus, dehydration, eating disorders, and depression [48,49]. In order to collect saliva from these patients, stimulation is necessary for rapid collection. 

**Figure 2:**
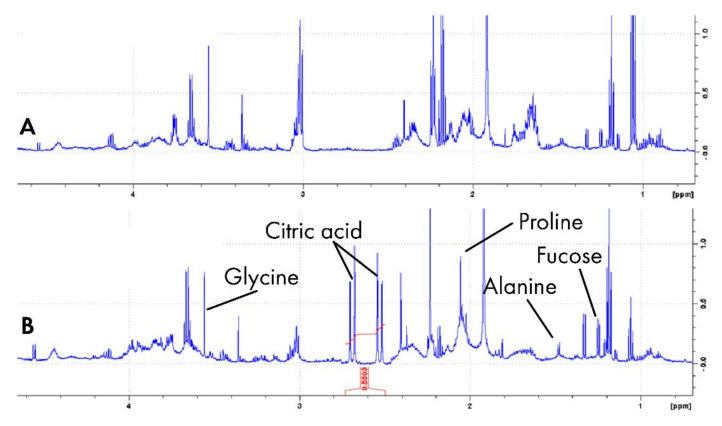
Spectral characteristics of human saliva (**A**) stimulated with paraffin (masticatory stimulation) and (**B**) by citric acid (gustatory stimulation). Citric acid added prominent peaks at around 2.50 and 2.70, which occur in a relatively unoccupied region of the spectrum [50].

The secretion of saliva can be stimulated by gustatory, olfactory, or masticatory stimuli. Paraffin wax is the most used masticatory stimulant, and citric acid is an example of a gustatory stimulus. The optimal sampling and storing procedures are important pre-analytically. Salivary stimulation to collect saliva samples is necessary for patients suffering from low salivary rates. Hence, we investigated the variations of metabolites with NMR for the two different salivary stimulations, e.g., masticatory stimulation (chewing with paraffin wax) and gustatory stimulation (citric acid) as presented in Figure 2.

The common preparation steps for salivary metabolomic analyses are centrifugation and storage in freezing temperatures. Of all the analysis techniques, NMR is a relatively novel and underutilized method for salivary analysis. In addition, saliva preparation for NMR is lacking a commonly agreed protocol. Centrifugation is an essential step to remove cell content and remove interference in the resulting spectrum. Repeated freezing and thawing are not to be found to affect the metabolic profile [51].

Inter-individual and inter-day variability in saliva samples are large and might affect the results more than variations in storage and handling. Microbial growth inhibitors such as NaN_3_ can be used to stabilize the samples, but in general, saliva samples are very stable. As for storage temperature, colder is better. Samples can be stored at −20 °C for up to 4 weeks without adverse effects to analysis [52].

## 6. Methods to Study Salivary Metabolites

The key technologies of salivary metabolomics are mass spectrometry (MS) in conjunction with either high-performance liquid chromatography (HPLC-MS) or two-dimensional gas chromatography (2DGC-MS), and nuclear magnetic resonance (NMR) spectroscopy (Table 1 and Table 2). 

Fourier-transform infrared (FTIR) spectroscopy utilizes infrared light for molecular composition analysis. Different molecular bonds absorb light at different wavelengths, and from spectral analysis, compounds and their concentrations can be identified. Photoacoustic spectroscopy (PAS) is a variation of this method and is primarily suitable for the measurement of gases. The weakness of these techniques is that they are sensitive to water, so proper drying before the measurement is required [53]. New methodologies to investigate patients’ health stages in the clinic are welcome. To study the saliva of oral surfaces, including mucosal pellicle, we need new technologies as presented by Hurskainen et al. [54]. Overall, more studies with this new method combining salivary metabolomics are needed before clinical use. 

Different bioinformatics tools can be used for metabolomics analyses. Spectral data can be analyzed with multivariate statistical analysis techniques such as principal component analysis (PCA) and partial least-squares regression (PLS). Simple univariate ANOVA and T-test methods can be used but are often insufficient when analyzing complex metabolic data [55]. 

Metabolic profiling can be done via targeted or untargeted analysis. The untargeted analysis attempts to analyze the whole salivary “fingerprint” and could yield information not initially considered as a testing outcome. The targeted analysis focuses on a certain subset of metabolites. This approach could lead to greater accuracy, but the metabolite subset must be selected carefully. Recently, machine learning algorithms have become more common [56], including a multiple logistic regression (MLR) model and an alternative decision tree (ADTree)-based machine learning method [35].

For practical clinical screening, large-scale analysis requires high-throughput methods. A method based on multisegment injection (MSI) combined with CE-MS was recently introduced. Using sample stacking to obtain sequential injection, it was possible to measure a series of salivary samples in one measurement run. [57]

Software packages are available for metabolomic analyses. For NMR spectral quantification, software such as PERCH (PERCH Solutions Ltd., Kuopio, Finland) has been used in our research [33]. Another open-source platform, PRIME, the Platform for RIKEN Metabolomics (prime.psc.riken.jp), assembles tools for metabolomics and transcriptomics. Moreover, MetaboAnalyst (5.0) is a freely available web application for complex metabolomic data analyses, visualization, and functional interpretation [58]. 

## 7. Discussion

The clinical interest in salivary metabolomics has been growing because saliva is easy to obtain using non-invasive methods and can be collected several times a day. Salivary metabolomics is a relatively new and developing research area that needs to be explored further for its optimal utilization in clinical implementations. As with most biological fluid, complications in the metabolic analysis occur as a consequence of the inhomogeneity of saliva’s composition and the high variability in saliva observed between individuals [38]. Furthermore, despite the methodological achievements in metabolomic analysis, the analysis of salivary metabolomics lacks standardized methods for saliva collecting, processing, and analyzing of saliva samples. These differences are a common challenge encountered in salivary research.

Metabolomic profiles of saliva are most probably a potential tool of choice for diagnosis, management, and follow-up of patients. With cross-sectional studies, we could not eliminate all factors, which are related to the salivary metabolite profile of certain diseases. Every human individual has their own salivary metabolic profile, so longitudinal study will give us information about the changes in different stages of certain diseases, predict disease progression, and the success of treatments. Consequently, during follow-up studies, we could find certain biomarkers for different specific diseases. Indeed, a validation step on a larger cohort of patients and controls is needed before clinical generalization of the method, such as Andörfer et al. presented [59].

The metabolite content of saliva and metabolic fingerprint in every subject is derived from the oral microbiome. Dysbiosis of oral microbes is a potential environmental factor both in oral and also in some cases of systemic diseases [19,60]. Metabolomic profiles of saliva observed in patients with different diseases may reflect a disease-associated oral microbiota, because most of the microorganisms, including viruses, modulate the metabolic profile of saliva. The metabolic fingerprints in different diseases and different oral conditions impact our understanding of the molecular mechanisms and metabolic pathways in the oral cavity. Because of the growing interest to move towards personalized treatments of many systemic diseases, salivary metabolomics may be a good tool for treatment planning. With the metabolic profile analysis of every patient, we could plan the personalized treatment and follow the treatment success.

The human oral cavity contains many different niches, which provide the appropriate space for the colonization of microorganisms, including bacteria, fungi, and viruses. Oral microorganisms are associated with several non-oral infections. The oral cavity is an important entry point for pathogens, including severe acute respiratory syndrome coronavirus 2 (SARS-CoV-2), which is found in saliva [61]. Coronavirus disease 2019 (COVID-19), which could be asymptomatic for three months, is an emerging infectious pandemic disease [62]. Thus, NMR-based metabolomics may be a prominent tool for the early-stage prediction diagnosis, and the frontline for the eradication/elimination of COVID-19 and the next pandemic [63].

Whole-mouth saliva is the most used in metabolic analysis. However, salivary components attach and concentrate on the oral mucosal surface as a mucosal pellicle. Differences in surfaces of the oral cavity, including teeth and oral mucosa, led to the development of physical and chemical sensors to analyze metabolites on these surfaces. To investigate metabolites of the mucosal pellicle, we need tools, such as physical or chemical properties of the sensor, to analyze the surface biofluid [54]. 

In the future, the salivary metabolic profile can serve as important knowledge regarding a patient’s health status, and according to the patient’s metabolic profiles, personalized treatment could be planned. Furthermore, saliva-derived biomarkers could be used to monitor the stage of a disease and treatment success. Further development of metabolomics for medical reasons, saliva samples should be collected at many standpoints from the same patient before and after treatment. Despite these advances, there is an important need for salivary metabolic analysis as a potential surrogate for other diagnostic methods. Saliva as a mirror of oral and systemic health, together with its metabolic investigations, will revolutionarily increase our knowledge regarding oral physiology and pathophysiology in the future. 

## Figures and Tables

**Table 1:** Recent salivary metabolomic studies of oral diseases.

Author, Year [Ref.]	Oral Diseases	Type of Saliva	Method
Aimetti et al. 2012 [9]	Periodontitis	WS	NMR
Barnes et al. 2014 [10]	Periodontitis and diabetes	NM	GC/MS and LC/MS
Marchesan et al. 2015 [11]	Periodontal diseases (dysbiosis)	WS	GC/MS and LC/MS
Kuboniwa et al. 2016 [12]	Periodontal inflammation	USWS	GC/MS
Rzeznik et al. 2017 [13]	Periodontitis	SWS	NMR
Romano et al. 2018 [14]	Periodontitis	USWS	NMR
Singh et al. 2019 [15]	Chronic periodontitis	WS	NMR
Liebsch et al. 2019 [16]	Periodontitis	SWS	UHPLC-MS/MS
Citterio et. al. 2020 [17]	Periodontitis after therapy	USWS	NMR
Kim et al. 2021 [18]	Periodontitis	SWS	NMR
Fidalgo et al. 2013 [20]	Children’s caries	USWS	NMR
Pereira et al. 2019 [21]	Children’s caries	USWS, SWS	NMR
Sugimoto et al. 2010 [22]	OSCC	WS	CE-TOF-MS
Wei et al. 2011 [23]	OSCC/leukoplakia	NM	LC-TOF-MS
Wang et al. 2014 [24]	OSCC	USWS	CE-MS
Lohavanichbutr et al. 2018 [25]	OSCC	NM	NMR, LC-MS
Ishikawa et al. 2019 [26]	OSCC (OED, PSOML)	USWS	CE-MS
Ishikawa et al. 2020 [27]	Lichen planus/OSCC	USWS	CE-MS
Yatsuoka et al. 2019 [28]	MRONJ	WS	CE-MS

NM = not mentioned; WS = whole saliva; USWS = unstimulated whole saliva; SWS = stimulated whole saliva; LC/MS = liquid chromatography-mass spectrometry; GC/MS = gas chromatography mass spectrometry; CE-TOF-MS = capillary electrophoresis time-of-flight mass spectrometry; UHPLC-MS/MS = ultra-high performance liquid chromatography and tandem mass spectrometry; NMR = NMR-spectroscopy; OSCC = oral squamous cell carcinoma; OED = oral epithelial dysplasia; PSOML = persistent suspicious oral mucosal lesions; MRONJ = medication-related osteonecrosis.

**Table 2:** A summary of studies investigating salivary metabolomics of systemic diseases.

Author, Year [Ref.]	Systemic Diseases	Type of Saliva	Method
Sugimoto et al. 2010 [22]	Breast and pancreatic cancer (OSCC)	WS	CE-TOF-MS
Xiao et al. 2012 [31]	Lung cancer	USWS	2D-DIGE-MS
Taware et al. 2018 [32]	HNC	USWS	GC/MS
Mikkonen et al. 2018 [33]	HNC	SWS	NMR
Grimaldi et al. 2018 [34]	Salivary gland (parotid) tumors	NM	NMR
Murata et al. 2019 [35]	Breast cancer	USWS	CE-TOF-MS
Xavier Assad et al. 2020 [36]	Breast cancer	NM	LC/MS
Mikkonen et al. 2013 [37]	Sjögren’s syndrome	SWS	NMR
Kageyama et al. 2015 [38]	Sjögren’s syndrome	USWS	GS/MS
Herrala et al. 2020 [39]	Sjögren’s syndrome	SWS	NMR
Barnes et al. 2014 [40]	Diabetes and periodontitis	NM	GC/MS and LC/MS
de Oliveira et al. 2016 [41]	Diabetes	WS	NMR
Figuera et al. 2016 [42]	Dementia	Collected at home	NMR
Symons et al. 2015 [43]	Cerebral palsy	USWS	NMR
Carro et al. 2017 [44]	AD and MCI	USWS	MALDI-TOF/MS
Yilmaz et al. 2017 [45]	AD and MCI	WS	NMR
Kumari et al. 2020 [46]	PD	USWS	NMR

USWS = unstimulated whole saliva; HNC = head and neck cancer; OSCC = oral squamous cell carcinoma; WS = whole saliva; CE-TOF-MS = capillary electrophoresis time-of-flight mass spectrometry; 2D-DIGE-MS = two-dimensional difference gel electrophoresis mass spectrometry; NM = not mentioned; GC/MS = gas chromatography-mass spectrometry; LC/MS = liquid chromatography-mass spectrometry; NMR = NMR-spectroscopy; PD = Parkinson’s disease; AD = Alzheimer’s disease; MCI = mild cognitive impairment; MALDI-TOF/TOF-MS = matrix-assisted laser-desorption ionization time-of-flight/time-of-flight mass spectroscopy; USWS = unstimulated whole saliva.

## References

[1] WHO International Programme on Chemical Safety Biomarkers in Risk Assessment: Validity and Validation. Environmental Health Criteria 222. http://www.inchem.org/documents/ehc/ehc/ehc222.htm.

[2] Bardow A., Lynge Pedersen A.M., Nauntofte B., Miles T.S., Nauntofte B., Svensson P. (2004). Saliva. Clinical Oral Physiology.

[3] Proctor G.B., Carpenter G.H. (2007). Regulation of salivary gland function by autonomic nerves. Auton. Neurosci. Basic Clin..

[4] Hanning C., Hanning M., Kensche A., Carpenter G. (2017). The mucosal pellicle- An underestimated factor in oral physiology. Arch. Oral Biol..

[5] Feller L., Altini M., Khammissa R.A.G., Chandran R., Bouckaert M., Lemmer J. (2013). Oral mucosal immunity. Oral Surg. Oral Med. Oral Pathol. Oral Radiol..

[6] Lynge Pedersen A.M., Belstrøm D. (2019). The role of natural salivary defences in maintaining a healthy oral microbiota. J. Dent..

[7] Proctor G.B. (2016). The physiology of salivary secretion. Periodontol. 2000.

[8] Pflughoeft K.J., Versalovic J. (2012). Human microbiome in health and disease. Ann. Rev. Pathol. Mech. Dis..

[9] Aimetti M., Cacciatore S., Graziano A., Tenori L. (2012). Metabonomic analysis of saliva reveals generalized chronic periodontitis signature. Metabolomics.

[10] Barnes V.M., Ciancio S.G., Shibly O., Xu T., Devizio W., Trivedi H.M., Guo L., Jönsson T.J. (2011). Metabolomics reveals elevated macromolecular degradation in periodontal disease. J. Dent. Res..

[11] Marchesan J.T., Morelli T., Moss K., Barros S.P., Ward M., Jenkins W., Aspiras M.B., Offenbacher S. (2015). Association of synergistetes and cyclodipeptides with periodontitis. J. Dent. Res..

[12] Kuboniwa M., Sakanaka A., Hashino E., Bamba T., Fukusaki E., Amano A. (2016). Prediction of periodontal inflammation via metabolic profiling of saliva. J. Dent. Res..

[13] Rzeznik M., Triba M.N., Levy P., Jungo S., Botosoa E., Duchemann B., Le Moyec L., Bernaudin J.F., Savarin P., Guez D. (2017). Identification of a discriminative metabolomic fingerprint of potential clinical relevance in saliva of patients with periodontitis using 1H nuclear magnetic resonance (NMR) spectroscopy. PLoS ONE.

[14] Romano F., Meoni G., Manavella V., Baima G., Tenori L., Cacciatore S., Aimetti M. (2018). Analysis of salivary phenotypes of generalized aggressive and chronic periodontitis through nuclear magnetic resonance-based metabolomics. J. Periodontol..

[15] Singh M.P., Saxena M., Saimbi C.S., Siddiqui M.H., Roy R. (2019). Post-periodontal surgery propounds early repair salivary biomarkers by 1 H NMR based metabolomics. Metabolomics.

[16] Liebsch C., Pitchika V., Pink C., Samietz S., Kastenmüller G., Artati A., Suhre K., Adamski J., Nauck M., Völzke H. (2019). The saliva metabolome in association to oral health status. J. Dent. Res..

[17] Citterio F., Romano F., Meoni G., Iaderosa G., Grossi S., Sobrero A., Dego F., Corana M., Berta C.N., Tenori L. (2020). Changes in the salivary metabolic profile of generalized periodontitis patients after non-surgical periodontal therapy: A metabolomic analysis using nuclear magnetic resonance spectroscopy. J. Clin. Med..

[18] Kim S., Kim H.J., Song Y., Lee A.H., Kim S., Chung J. (2021). Metabolic phenotyping of saliva to identify possible biomarkers of periodontitis using proton nuclear magnetic resonance. J. Clin. Periodontol..

[19] Pietiäinen M., Liljestrand J.M., Kopra E., Pussinen P.J. (2018). Mediators between oral dysbiosis and cardiovascular diseases. Eur. J. Oral Sci..

[20] Fidalgo T.K.S., Freitas-Fernandes L.B., Angeli R., Muniz A.M.S., Gonsalves E., Santos R., Nadal J., Almeida F.C.L., Valente A.P., Souza I.P.R. (2013). Salivary metabolite signatures of children with and without dental caries lesions. Metabolomics.

[21] Pereira J.L., Duarte D., Carneiro T.J., Ferreira S., Cunha B., Soares D., Costa A.L., Gil A.M. (2019). Saliva NMR metabolomics: Analytical issues in pediatric oral health research. Oral Dis..

[22] Sugimoto M., Wong D.T., Hirayama A., Soga T., Tomita M. (2010). Capillary electrophoresis mass spectrometry-based saliva metabolomics identified oral, breast and pancreatic cancer-specific profiles. Metabolomics.

[23] Wei J., Xie G., Zhou Z., Shi P., Qiu Y., Zheng X., Chen T., Su M., Zhao A., Jia W. (2011). Salivary metabolite signatures of oral cancer and leukoplakia. Int. J. Cancer.

[24] Wang Q., Gao P., Wang X., Duan Y. (2014). The early diagnosis and monitoring of squamous cell carcinoma via saliva metabolomics. Sci Rep..

[25] Lohavanichbutr P., Zhang Y., Wang P., Gu H., Nagana Gowda G.A., Djukovic D., Buas M.F., Raftery D., Chen C. (2018). Salivary metabolite profiling distinguishes patients with oral cavity squamous cell carcinoma from normal controls. PLoS ONE.

[26] Ishikawa S., Wong D.T.W., Sugimoto M., Gleber-Netto F.O., Li F., Tu M., Zhang Y., Akin D., Iino M. (2019). Identification of salivary metabolites for oral squamous cell carcinoma and oral epithelial dysplasia screening from persistent suspicious oral mucosal lesions. Clin. Oral Investig..

[27] Ishikawa S., Sugimoto M., Edamatsu K., Sugano A., Kitabatake K., Iino M. (2020). Discrimination of oral squamous cell carcinoma from oral lichen planus by salivary metabolomics. Oral Dis..

[28] Yatsuoka W., Ueno T., Miyano K., Uezono Y., Enomoto A., Kaneko M., Ota S., Soga T., Sugimoto M., Ushijima T. (2019). Metabolomic profiling reveals salivary hypotaurine as a potential early detection marker for medication-related osteonecrosis of the jaw. PLoS ONE.

[29] Washio J., Takahashi N. (2016). Metabolomic studies of oral biofilm, oral cancer, and beyond. Int. J. Mol. Sci..

[30] Toan N.K., Ahn S.G. (2021). Aging-Related Metabolic Dysfunction in the Salivary Gland: A Review of the Literature. Int. J. Mol. Sci..

[31] Xiao H., Zhang L., Zhou H., Lee J.M., Garon E.B., Wong D.T.W. (2012). Proteomic analysis of human saliva from lung cancer patients using two-dimensional difference gel electrophoresis and mass spectrometry. Mol. Cell. Proteom..

[32] Taware R., Taunk K., Pereira J.A.M., Shirolkar A., Soneji D., Câmara J.S., Nagarajaram H.A., Rapole S. (2018). Volatilomic insight of head and neck cancer via the effects observed on saliva metabolites. Sci. Rep..

[33] Mikkonen J.J.W., Singh S.P., Akhi R., Salo T., Lappalainen R., González-Arriagada W.A., Lopes M.A., Kullaa A.M., Myllymaa S. (2018). Potential role of nuclear magnetic resonance spectroscopy to identify salivary metabolite alterations in patients with head and neck cancer. Oncol. Lett..

[34] Grimaldi M., Palisi A., Rossi G., Stillitano I., Faiella F., Montoro P., Rodriquez M., Palladino R., D’Ursi A.M., Romano R. (2018). Saliva of patients affected by salivary gland tumour: An NMR metabolomics analysis. J. Pharm. Biomed. Anal..

[35] Murata T., Yanagisawa T., Kurihara T., Kaneko M., Ota S., Enomoto A., Tomita M., Sugimoto M., Sunamura M., Hayashida T. (2019). Salivary metabolomics with alternative decision tree-based machine learning methods for breast cancer discrimination. Breast Cancer Res Treat..

[36] Xavier Assad D., Acevedo A.C., Cançado Porto Mascarenhas E., Costa Normando A.G., Pichon V., Chardin H., Neves Silva Guerra E., Combes A. (2020). Using an untargeted metabolomics approach to identify salivary metabolites in women with breast cancer. Metabolites.

[37] Mikkonen J.J.W., Herrala M., Soininen P., Lappalainen R., Tjäderhane L., Seitsalo H., Niemelä R., Salo T., Kullaa A.M., Myllymaa S. (2013). Metabolic Profiling of Saliva in Patients with Primary Sjögren’s syndrome. Metabolomics.

[38] Kageyama G., Saegusa J., Irino Y., Tanaka S., Tsuda K., Takahashi S., Sendo S., Morinobu A. (2015). Metabolomics analysis of saliva from patients with primary Sjögren’s syndrome. Clin. Exp. Immunol..

[39] Herrala M., Mikkonen J.J.W., Pesonen P., Lappalainen R., Tjäderhane L., Niemelä R.K., Seitsalo H., Salo T., Myllymaa S., Kullaa A.M. (2020). Metabolomics analysis of saliva from patients with primary Sjögren’s syndrome. J. Oral Dis..

[40] Barnes V.M., Kennedy A.D., Panagakos F., Devizio W., Trivedi H.M., Jönsson T., Guo L., Cervi S., Scannapieco F.A. (2014). Global metabolomic analysis of human saliva and plasma from healthy and diabetic subjects, with and without periodontal disease. PLoS ONE.

[41] De Oliveira L.R., Martins C., Fidalgo T.K., Freitas-Fernandes L.B., de Oliveira Torres R., Soares A.L., Almeida F.C., Valente A.P., de Souza I.P. (2016). Salivary Metabolite Fingerprint of Type 1 Diabetes in Young Children. J. Proteome Res..

[42] Figueira J., Jonsson P., Adolfsson A.N., Adolfsson R., Nyberg L., Öhman A. (2016). NMR analysis of the human saliva metabolome distinguishes dementia patients from matched controls. Mol. Biosyst..

[43] Symons F.J., ElGhazi I., Reilly B.G., Barney C.C., Hanson L., Panoskaltsis-Mortari A., Armitage I.M., Wilcox G.L. (2015). Can biomarkers differentiate pain and no pain subgroups of nonverbal children with cerebral palsy? A preliminary investigation based on noninvasive saliva sampling. Pain Med..

[44] Carro E., Bartolomé F., Bermejo-Pareja F., Villarejo-Galende A., Molina J.A., Ortiz P., Calero M., Rabano A., Cantero J.L., Orive G. (2017). Early diagnosis of mild cognitive impairment and Alzheimer’s disease based on salivary lactoferrin. Alzheimers Dement.

[45] Yilmaz A., Geddes T., Han B., Bahado-Singh R.O., Wilson G.D., Imam K., Maddens M., Graham S.F. (2017). Diagnostic biomarkers of Alzheimer’s disease as identified in saliva using ^1^H NMR-based metabolomics. J. Alzheimers Dis..

[46] Kumari S., Goyal V., Kumaran S.S., Dwivedi S.N., Srivastava A., Jagannathan N.R. (2020). Quantitative metabolomics of saliva using proton NMR spectroscopy in patients with Parkinson’s disease and healthy controls. Neurol. Sci..

[47] Belstrøm D., Holmstrup P., Bardow A., Kokaras A., Fiehn N.E., Paster B.J. (2016). Comparative analysis of bacterial profiles in unstimulated and stimulated saliva samples. J. Oral Microbiol..

[48] Pedersen A.M.L., Sørensen C.E., Proctor G.B., Carpenter G.H., Ekström J. (2018). Salivary secretion in health and disease. J. Oral Rehabil..

[49] Pina G.M.S., Mota Carvalho R., Silva B.S.F., Almeida F.T. (2020). Prevalence of hyposalivation in older people: A systematic review and meta-analysis. Gerodontology.

[50] Mikkonen J.J.W. (2019). Infrared and Nuclear Magnetic Resonance Spectroscopic Methods for Salivary Analysis. Ph.D. Thesis.

[51] Gardner A., Parkes H.G., Carpenter G.H., So P.W. (2018). Developing and standardizing a protocol for quantitative proton nuclear magnetic resonance (^1^H NMR) spectroscopy of saliva. J. Proteome Res..

[52] Duarte D., Castro B., Pereira J.L., Marques J.F., Costa A.L., Gil A.M. (2020). Evaluation of saliva stability for NMR metabolomics: Collection and handling protocols. Metabolites.

[53] Mikkonen J.J.W., Raittila J., Rieppo L., Lappalainen R., Kullaa A.M., Myllymaa S. (2016). Fourier transform infrared spectroscopy and photoacoustic spectroscopy for saliva analysis. Appl. Spectrosc..

[54] Hurskainen M., Sarin J.K., Myllymaa S., González-Arriagada G.A., Kullaa A., Lappalainen R. (2021). Feasibility of near-infrared spectroscopy for identification of L-fucose and L-proline—Towards detecting biomarkers from saliva. Metabolites.

[55] Trezzi J.P., Jäger C., Galozzi S., Barkovits K., Marcus K., Mollenhauer B., Hiller K. (2017). Metabolic profiling of body fluids and multivariate data analysis. MethodsX.

[56] Blekherman G., Laubenbacher R., Cortes D.F., Mendes P., Torti F.M., Akman S., Torti S.V., Shulaev V. (2011). Bioinformatics tools for cancer metabolomics. Metabolomics.

[57] Igarashi K., Ota S., Kaneko M., Hirayama A., Enomoto M., Katumata K., Sugimoto M., Soga T. (2021). High-throughput screening of salivary polyamine markers for discrimination of colorectal cancer by multisegment injection capillary electrophoresis tandem mass spectrometry. J. Chromatogr. A.

[58] Pang Z., Chong J., Zhou G., de Lima Morais D.A., Chang L., Barrette M., Gauthier C., Jacques P.É., Li S., Xia J. (2021). MetaboAnalyst 5.0: Narrowing the gap between raw spectra and functional insights. Nucleic Acids Res..

[59] Andörfer L., Holtfreter B., Weiss S., Matthes R., Pitchika V., Schmidt C.O., Samietz S., Kastenmüller G., Nauck M., Völker U. (2021). Salivary metabolites associated with a 5-year tooth loss identified in a population-based setting. BMC Med..

[60] Graves D.T., Corrêa J.D., Silva T.A. (2019). The oral microbiota is modified by systemic diseases. J. Dent. Res..

[61] Xu R., Cui B., Duan X., Zhang P., Zhou X., Yuan Q. (2020). Saliva: Potential diagnostic value and transmission of 2019-nCoV. Int. J. Oral Sci..

[62] Bordea I.R., Xhajanka E., Candrea S., Bran S., Onișor F., Inchingolo A.D., Malcangi G., Pham V.H., Inchingolo A.M., Scarano A. (2020). Coronavirus (SARS-CoV-2) Pandemic: Future Challenges for Dental Practitioners. Microorganisms.

[63] Costa Dos Santos Junior G., Pereira C.M., Kelly da Silva Fidalgo T., Valente A.P. (2020). Saliva NMR-based metabolomics in the war against COVID-19. Anal. Chem..

